# Whole Genome Identification and Biochemical Characteristics of the *Tilletia horrida* Cytochrome P450 Gene Family

**DOI:** 10.3390/ijms251910478

**Published:** 2024-09-28

**Authors:** Yafei Wang, Yan Shi, Honglian Li, Senbo Wang, Aijun Wang

**Affiliations:** College of Plant Protection, Henan Agricultural University, Zhengzhou 450002, China; shiyan00925@126.com (Y.S.); honglianli@sina.com (H.L.); 16639571007@163.com (S.W.)

**Keywords:** *Tilletia horrida*, rice kernel smut, cytochrome P450 (CYP) enzymes, expression pattern

## Abstract

Rice kernel smut caused by the biotrophic basidiomycete fungus *Tilletia horrida* causes significant yield losses in hybrid rice-growing areas around the world. Cytochrome P450 (CYP) enzyme is a membrane-bound heme-containing monooxygenase. In fungi, CYPs play a role in cellular metabolism, adaptation, pathogenicity, decomposition, and biotransformation of hazardous chemicals. In this study, we identified 20 *CYP* genes based on complete sequence analysis and functional annotation from the *T. horrida* JY-521 genome. The subcellular localization, conserved motifs, and structures of these 20 *CYP* genes were further predicted. The *ThCYP* genes exhibit differences in gene structures and protein motifs. Subcellular localization showed that they were located in the plasma membrane, cytoplasm, nucleus, mitochondria, and extracellular space, indicating that they had multiple functions. Some cis-regulatory elements related to stress response and plant hormones were found in the promoter regions of these genes. Protein–protein interaction (PPI) analysis showed that several ThCYP proteins interact with multiple proteins involved in the ergosterol pathway. Moreover, the expression of 20 *CYP* genes had different responses to different infection time points and underwent dynamic changes during *T. horrida* JY-521 infection, indicating that these genes were involved in the interaction with rice and their potential role in the pathogenic mechanism. These results provided valuable resources for elucidating the structure of *T. horrida* CYP family proteins and laid an important foundation for further research of their roles in the pathogenesis.

## 1. Introduction

Cytochrome P450 (CYP) is a protein family of mixed functional oxidoreductases, first discovered in the early 1960s [1]. CYPs are widely present in the biological world, and genome sequencing projects have revealed that their numbers are constantly increasing [2]. CYPs are composed of enzymes from a large superfamily with unique folding characteristics. These enzymes are located in the cell membrane or cytoplasm and participate in different reactions, contributing to the transport, metabolism, and catabolism of organic substrates [2,3]. CYPs have a wide range of substrates, catalyze different reactions, and possess powerful functions. CYPs can catalyze the degradation of polycyclic aromatic hydrocarbons, insecticides, and other organic compounds [4,5,6,7,8,9,10].

In fungi, CYPs play a role in cellular metabolism, adaptation, pathogenicity, decomposition, and biotransformation of hazardous chemicals. Fungal CYPs are widely distributed in different phyla and participate in a series of cellular processes, including secondary metabolite biosynthesis, ergosterol biosynthesis, and exogenous biotransformation [11]. Despite sharing conserved motifs, the sequence similarity between CYPs is low [12]. CYP monooxygenases not only catalyze hydroxylation reactions but also catalyze epoxidation, dehalogenation, decarboxylation, demethylation, denitrification, desulfurization, and desaturation on a wide range of substrates [13]. Fungal CYPs play an important regulatory role in the interaction between fungi and hosts, affecting the outcomes of fungal infections and host immune responses. These enzymes catalyze redox reactions within fungal cells and participate in various biosynthetic pathways and metabolic processes. In the interaction between fungi and hosts, CYPs can affect the infectivity, toxicity, and pathogenicity of fungi to hosts. They can metabolize toxins, pathogenic factors, or antibiotics and regulate the adaptability and survival ability of fungi to their hosts [14,15]. Fungal CYPs can also interact with metabolic pathways within host cells, affecting the host’s immune response and resistance to fungi [14]. According to previous reports, the ability of white rot fungi to degrade lignin and lignin-like substances is closely related to their CYP450 [11].

*Tilletia horrida* is an important pathogenic fungus of rice (*Oryza sativa* L.) [16]. This disease is widely distributed in rice-producing countries in Asia, America, and Africa and most rice-growing areas in China. Especially after the introduction of hybrid rice, the incidence rate of rice kernel smut (RKS) in the rice area further increased, which seriously affected the rice yield and seed quality. In hybrid rice cultivation, this disease can cause devastating damage to the production and quality of male sterile rice varieties [17,18]. In nature, *T. horrida* mainly exists in the form of spores and has a strong ability to resist adversity. Under natural conditions, they can survive for more than one year [19]. In the infection stage, the key feature of this fungus is that it mainly infects rice grains from the stigma in the early stages of flowering, forming a black spore sac filled with spores [20].

In recent years, with the development of molecular biology and genomic technologies, researchers have begun to pay attention to the genome structure, identification of pathogenic genes, and disease resistance mechanisms of important pathogenic fungi in rice. The whole genome sequencing of *T. horrida* JY-521 laid the foundation for molecular biology research and provided conditions for analyzing genes and proteins related to the pathogenesis [21,22]. However, the pathogenesis of *CYP* genes in the pathogenic fungi is still unclear and requires further research. This study identified all *CYP* family genes coding proteins by searching the genome of *T. horrida* JY-521 and analyzed these genes through whole genome identification and biochemical characterization. Phylogenetic analysis is used to study the interrelationships among *CYP* genes, while Multiple Em for Motif Elicitation (MEME) and Gene Structure Display Server (GSDS) are used to predict the conserved motifs and structures of *CYP* genes. We analyzed the relative expression of *CYP* genes during infection and constructed their protein–protein interaction network. *CYP* genes may play an important role in the pathogenesis of *T. horrida*. This study promoted our deeper understanding of *CYP* genes in *T. horrida* and provides ideas for further research on their roles in the pathogenesis of *T. horrida*.

## 2. Results

### 2.1. Identification and Analysis of Physicochemical Properties of ThCYP Genes

Cytochrome P450 genes were identified in the P450 database with an e-value of ≤1 × 10^−5^ (http://drnelson.uthsc.edu/cytochromeP450.html (accessed on 23 September 2024)) [22,23]. A total of 20 *CYP* family genes (named *ThCYP1*–*ThCYP20*) were detected in the *T. horrida* JY-521 genome. Among them, most ThCYPs belong to the superfamily CYP4, while four ThCYPs belong to the superfamily CYP3 [23]. Table 1 lists their detailed characteristics, including protein length, molecular weight, isoelectric point, and grand average of hydropathicity (GRAVE). The length of ThCYP family proteins ranges from 496 to 1183 amino acids (aa), and the predicted molecular weight is between 55.82 and 130.99 kDa. These results indicate that ThCYPs have a wide range of isoelectric points and molecular weights. These proteins are divided into three acidic proteins (PI value < 6.5), fourteen alkaline proteins (PI value > 7.5), and three neutral proteins (6.5 < PI value < 7.5). The grand average of hydropathicity ranges from −0.375 to 0.058, with ThCYP4 and ThCYP7 having grand averages of hydropathicity of 0.031 and 0.058, respectively, while the rest of the grand averages of hydropathicity are less than zero. The results indicate that the vast majority of ThCYP proteins are hydrophilic. In addition, we also predicted the subcellular localization of ThCYP proteins using tools on the PSORT website, and the results showed that ThCYP3, ThCYP10, ThCYP15, and ThCYP18 were localized in the cytoplasm; ThCYP1, ThCYP4, ThCYP6, ThCYP8, ThCYP9, ThCYP12, ThCYP16, and ThCYP19 were localized in the mitochondrion; ThCYP2, ThCYP5, ThCYP7, and ThCYP14 were localized in the plasma membrane; ThCYP13, ThCYP17, and ThCYP20 were localized in the extracellular space; and ThCYP11 was localized in the nucleus (Table 1).

### 2.2. Phylogenetic Relationships

**Figure 1:**
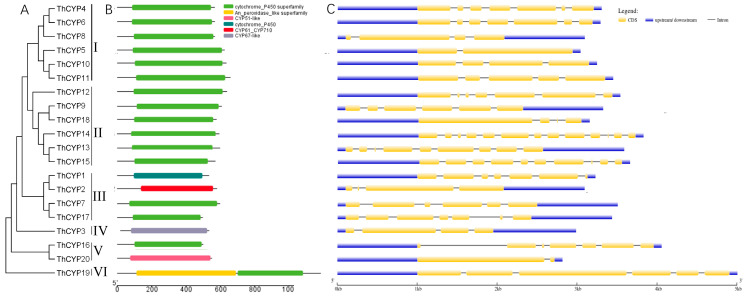
Phylogenetic relationship, domains, and gene structure analysis of ThCYPs. Note: (**A**) Phylogenetic tree of ThCYP proteins. (**B**) Domain analysis of ThCYPs. (**C**) Exon–intron structures of ThCYPs. The yellow box represents the exons; the black line represents the introns; the blue box represents the non-coding area. The horizontal axis represents the full length of the sequences.

To understand the phylogenetic relationship of CYP family proteins, a phylogenetic tree was constructed based on the protein sequences of 20 CYPs in *T. horrida* JY-521 (Table S1). According to the MEGA5 analysis results, they are divided into six groups, with groups I, II, III, IV, V, and VI having 6, 6, 4, 1, 2, and 1 member, respectively (Figure 1A). The close distribution of groups I and II on the phylogenetic tree indicates a close genetic relationship between these two groups. The evolutionary tree distance between group VI and group I is far, indicating a poor genetic relationship between these two groups.

### 2.3. Sequence and Structure Analysis of ThCYPs

To understand the characteristics of the *T. horrida* JY-521 CYP family, we analyzed the structural features of all *ThCYP* genes (Figure 1B,C and Table S1). According to the result, the *ThCYP* genes have significantly different gene structures. Domain analysis clearly indicates that most *ThCYPs* have a cytochrome P450 superfamily domain (Figure 1B). In addition, *ThCYP2* has the CYP61/CYP710 domain, and *ThCYP3* has the CYP67-like domain. As shown in Figure 1C, the 20 *ThCYPs* contain 1 to 13 exons and 1 to 12 introns, with the number of exons and introns varying depending on the genes. Among them, *ThCYP14* has the highest number of exons and introns, with 13 and 12, respectively, while *ThCYP20* has only 1 intron. This suggests that the *ThCYP* genes may have undergone events of loss and acquisition of exons and introns during evolution. Furthermore, the result shows that genes with similar structures are usually clustered in the same category.

**Figure 2:**
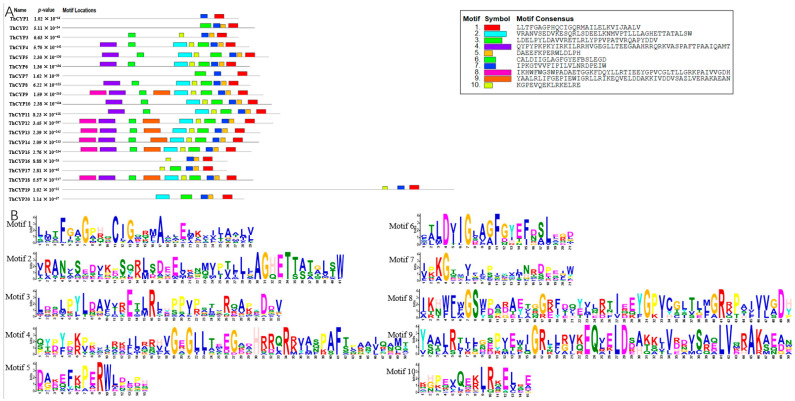
The motifs of ThCYPs predicted by MEME. (**A**) Distinct colored boxes denoting the various conserved motifs having differed sizes and sequences. (**B**) Sequence logo conserved motif of ThCYP proteins.

To understand the function of the *ThCYP* genes, we used the website Multiple Em for Motif Elicitation (MEME) to evaluate the conserved motifs of the ThCYP proteins. A total of 10 motifs in ThCYP proteins were ultimately predicted (Figure 2). Motif 1 and motif 7 are the most common motifs, found in all ThCYP proteins. Motif 5 was detected in 17 ThCYP proteins, motif 10 was detected in 16 ThCYP proteins, and motif 3 was detected in 15 ThCYP proteins. Motifs 2 and 6 were detected in 13 ThCYP proteins. Motif 4 was detected in 12 ThCYP proteins. Motifs 8 and 9 were detected in six ThCYP proteins. In addition, six ThCYP proteins contain 10 motifs simultaneously. Generally speaking, the conserved motifs of these proteins within the same group are similar in composition, indicating that these ThCYPs may have similar functions. In addition, the three-dimensional (3D) structure of ThCYP proteins was predicted using the phyre2 online network tool to gain a better understanding of the biological function of *ThCYP* genes in rice (Figure S1).

### 2.4. Promoter Sequence Analysis of ThCYP Gene Family

**Figure 3:**
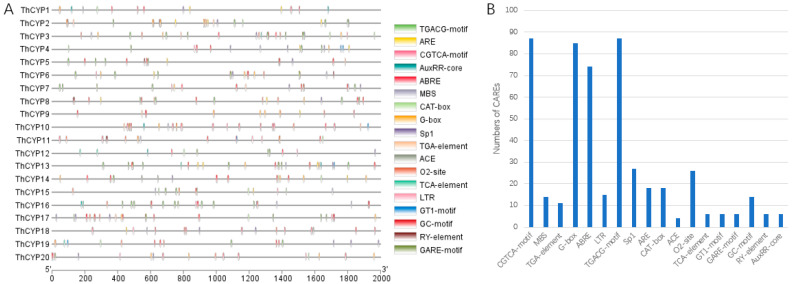
Analysis of cis-acting elements in the promoter regions of *ThCYP*s. Note: (**A**) Different colored boxes represent different cis-acting elements. (**B**) Most frequently predicted CAREs in the *ThCYP* promoter regions.

Using the PlantCARE online network server, the 2000 bp upstream sequences of *ThCYP* genes were detected to understand the exact roles of these genes (Table S1). Based on this study, the promoter region of the ThCYP gene included many cis-elements related to development, pathogenicity, and stress response (Figure 3A and Table S2). Further analysis results showed that the promoter region of the *ThCYP* gene mainly included MeJA responsive elements (TGACG-motif and CGTCA-motif), light responsive elements (G-box, GT1 motif and Sp1, ACE), abscisic acid responsive elements (ABRE), zein metabolism regulation responsive elements (O2-site), low-temperature responsive elements (LTR), auxin response elements (TGA-element and AuxRR-core), gibberellin responsive elements (GARE-motif), and drought responsive elements (MBS). Among them, MeJA has the highest number of responsive elements (174), followed by photoresponsive elements (122), abscisic acid responsive elements (70), and zein metabolism regulatory responsive elements (24) (Figure 3B and Table S2).

The results of this study indicate that ThCYPs play an indispensable role in fungal growth, pathogenicity, and response to various stress conditions. In addition, ThCYPs also include CAREs associated with anaerobic induction (ARE), meristem expression (CAT-box), hypoxia-specific induction (GC-motif), salicylic acid (TCA-element), and seed-specific regulation (RY-element) (Figure 3 and Table S2). It is worth noting that LTR and MBS belong to stress response elements. ARE, CGTCA-motif, TGACG-motif, TCA, ABRE, TGA, and AuxRR-core are all hormone response elements. The CAREs described in the *ThCYP* gene family confirm that ThCYPs may be associated with multiple biological processes. Therefore, these results provide crucial information for understanding the complex regulatory network of the *ThCYP* gene family in different developmental processes, pathogenic processes, and multifactorial stress.

### 2.5. Gene Ontology Enrichment Analysis of the ThCYP Genes

**Figure 4:**
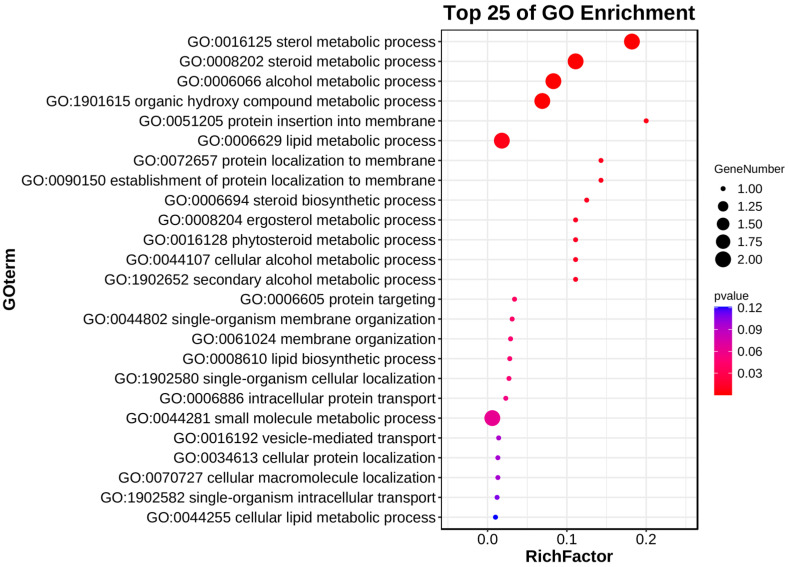
Gene ontology enrichment analysis of *ThCYP* genes.

Gene ontology (GO) enrichment analysis helps to understand the function of genes by comparing protein sequences with known functions of species proteins, and *ThCYP* family genes are enriched here (Figure 4 and Table S3). The GO enrichment analysis results showed that *ThCYP2, ThCYP3,* and *ThCYP20* enriched in the metabolic process (GO:0008152). *ThCYP2* and *ThCYP20* also enriched in the sterol metabolic process (GO:0016125), steroid metabolic process (GO:0008202), alcohol metabolic process (GO:0006066), organic hydroxy compound metabolic process (GO:1901615), and lipid metabolic process (GO:0006629). The conclusion drawn from these findings is that some *ThCYP* genes may play a role in the metabolic process, especially in sterol metabolism, which further affects fungal drug resistance, pathogenicity, and biological adaptability.

### 2.6. The Response of ThCYP Family Genes during T. horrida Infection in Rice

**Figure 5:**
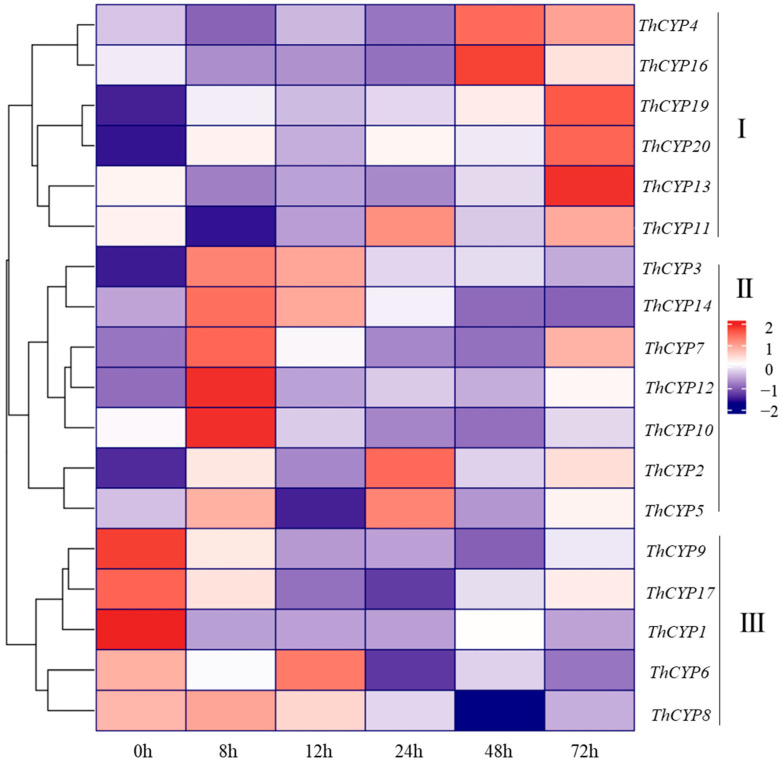
The expression level of *ThCYP*s based on RNA-seq data. Note: Different colors represent different FPKM values. Red and blue indicate high and low expression levels of *ThCYP*s, respectively.

**Figure 6:**
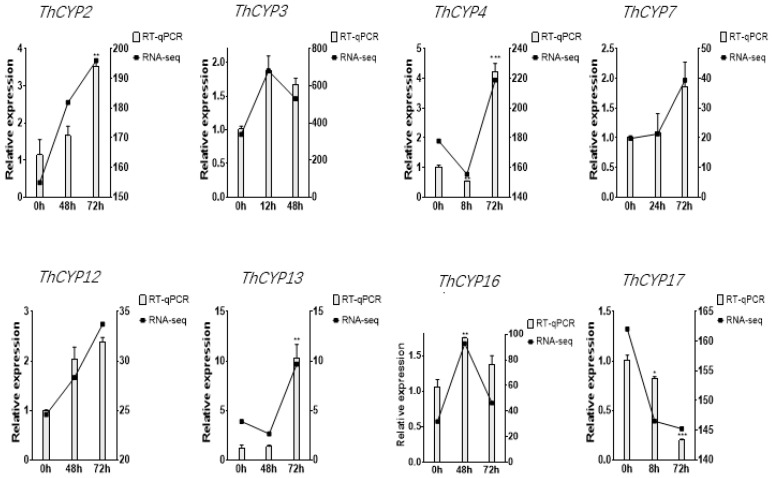
Expression profiles of *ThCYP*s by RT-qPCR. Note: Bars represent the mean values of three technical replicates ± SE, Student’s *t*-test (*n* = 3, * *p* < 0.05, ** *p* < 0.01, *** *p* < 0.001). The histograms represent RT-qPCR data, and line charts represent FPKM data.

In order to search for *ThCYP* family genes involved in the pathogenesis of *T. horrida*, the relative expression of 20 *CYP* genes in rice infected with *T. horrida* JY-521 was studied using transcriptome sequencing data from the previous research [21]. We generated a heatmap based on Fragments Per Kilobase of exon model per Million mapped fragments (FPKM) values of 20 *CYP* family genes, showing their expression profiles at 6 time points (0 h, 8 h, 12 h, 24 h, 48 h, and 72 h) after inoculation (Figure 5). The result shows that the expression patterns of 20 *CYP* family genes can be divided into three maps based on the trend changes at six time points. Map I, which includes six *CYP* family genes (*ThCYP4*, *ThCYP11*, *ThCYP13*, *ThCYP16*, *ThCYP19*, and *ThCYP20*), showed significant up-regulation of transcription levels during infection. Map III, which includes five *CYP* family genes (*ThCYP1*, *ThCYP6*, *ThCYP8, ThCYP9*, and *ThCYP17*), showed significant down-regulation. In addition, it is worth noting that map II contains seven *CYP* family genes (*ThCYP2*, *ThCYP3*, *ThCYP5*, *ThCYP7*, *ThCYP10*, *ThCYP12*, and *ThCYP14*), which exhibit high transcription levels at one time point during the early stages of infection. It is speculated that these *ThCYP* family genes may play a crucial role in the pathogenicity. To verify the accuracy of transcriptome data, specific primers of eight *ThCYP* genes were designed for RT-qPCR analysis (Table S4). The results of RT-qPCR analysis showed that the expression of *ThCYP*s was dynamically changing during the infection process, and the change trend of different genes was significantly different. For example, *ThCYP2*, *ThCYP7*, and *ThCYP12* were significantly up-regulated at 72 h, while *ThCYP17* was significantly down-regulated at 72 h. The expressions of *ThCYP4* and *ThCYP13* were down-regulated and then up-regulated during infection, while the expressions of *ThCYP3* and *ThCYP16* were up-regulated and then down-regulated during infection. The expression trends of these genes were basically consistent with the transcriptome data, indicating the reliability of the transcriptome data analysis results (Figure 6).

### 2.7. Protein–Protein Interaction Network

**Figure 7:**
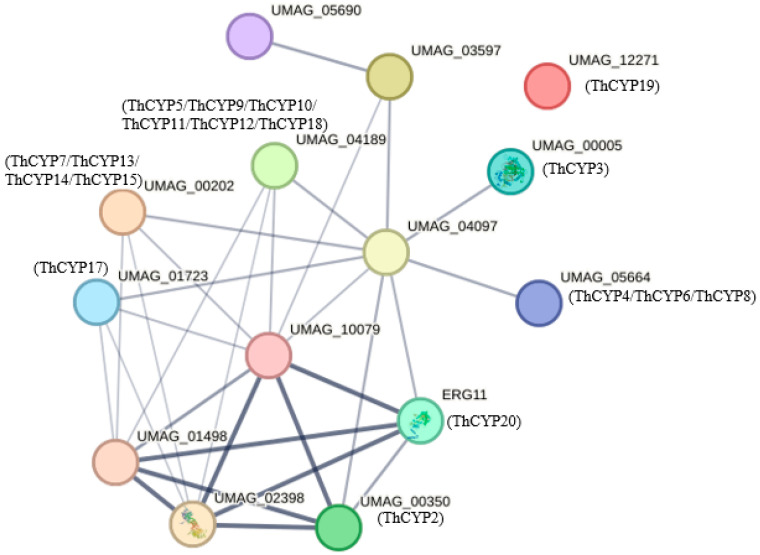
Protein–protein interaction network of ThCYP family proteins. Note: UMAG_01498: putative sterol C-24 reductase; UMAG_02398: squalene monooxygenase; UMAG_10079: putative lanosterol synthase; ERG11: lanosterol 14-alpha demethylase. The unmarked proteins in the picture are unknown proteins.

In order to understand the functional characteristics of ThCYP family proteins, the protein–protein interaction (PPI) network was analyzed using STRING v12.0. PPI predicted 14 nodes and 30 edges, with an average node degree of 4.29 and an average local clustering coefficient of 0.626 (PPT enrichment *p*-value: 2.46 × 10^−13^). We set the confidence level of the medium to 0.4 to predict functional chaperone proteins. Multiple functional partner proteins were predicted, including sterol C-24 reductase (UMAG01498), squalene monooxygenase (UMAG02398), and lanosterol synthase (UMAG10079) (Figure 7). In addition, sterol C-24 reductase (UMAG01498), squalene monooxygenase (UMAG02398), lanosterol synthase (UMAG10079), ThCYP2(UMAG000350), and ThCYP20 (ERG11) form the core of the interaction. These functional partner proteins may work together with ThCYPs to perform biological functions and play important roles in metabolic processes. Based on the PPI results and the function of partner proteins, it can be inferred that partner proteins may be involved in the interaction between plants and pathogens and contribute to pathogen infection.

## 3. Discussion

Fungal cytochrome P450 enzymes play an important role in the pathogenic process of fungi [14,24]. These enzymes are involved in key metabolic pathways such as fungal toxin synthesis, pathogenic factors, and antifungal drug resistance, which affect the ability of fungi to infect hosts [25,26,27]. By regulating the synthesis and degradation of fungal metabolites, fungal cytochrome P450 enzymes can affect the toxicity, adaptability, and survival ability of fungi, thereby affecting their pathogenicity to hosts [25]. During the pathogenic process, fungal cytochrome P450 enzymes can help fungi produce toxic reactions to hosts, inhibit host immune responses, and promote fungal growth and spread [14]. In addition, these enzymes can also participate in the adaptive response of fungi to the external environment, improving their survival ability [9,10,14].

In this study, we identified a total of 20 *CYP* family genes in *T. horrida* and used bioinformatic analysis to analyze their gene structures and protein motifs. According to the functional domains of 20 CYP family proteins, they are classified into different CYP subfamilies. In the CYP superfamily of fungi, there are several families that have attracted much attention, such as the structurally and functionally conserved fungal CYP family CYP61 [28,29]. Ergosterol is a unique sterol found in pathogenic fungi, and the ergosterol pathway is a target for many important antifungal drugs [24,30]. In fungi, the *CYP61* gene encodes C-22 sterol desaturase and is involved in the late stage of the ergosterol pathway [11,31]. In this study, ThCYP7 has the CYP61 domain, and it may play an important role in the ergosterol pathway. ThCYP3 has the CYP67 domain, and most ThCYPs have a cytochrome P450 superfamily domain. These results suggest that ThCYPs may play a role in different metabolic processes. Further research is needed to validate the functions of ThCYP3, and ThCYP7 and elucidate their regulatory and molecular mechanisms. In addition, these findings combined with 3D structural analysis of ThCYP proteins will help further elucidate the molecular mechanisms by which ThCYPs participate in fungal signaling pathways.

In addition, phylogenetic analysis of 20 CYP family proteins showed that ThCYP19 had distant relationships with other ThCYPs. Phylogenetic analysis revealed the relationships and evolutionary history among these proteins. The functions of these proteins in *T. horrida* JY-521 have hardly been reported, and further attention is needed to their pathogenicity and infection process. The diversity of fungal CYPs also provides more possibilities for their application. Due to extensive gene duplication events or gene recombination, the multifunctional properties of CYP monooxygenase may have originated from different ancestors [32,33]. The evolution of fungal CYPs may be driven in part by survival strategies aimed at providing a superior metabolic system for degrading exogenous chemicals, possibly involving plant-related compounds such as lignin and its derivatives [34].

The number of exons and introns plays a crucial role in altering gene function during evolution [35,36], and genes with fewer introns can be activated more quickly in environmental challenges [37]. In many microorganisms, the exon–intron distribution pattern in genes encoding proteins exhibits discrete features [38]. The gene structure and conserved motifs of the same group in this study are similar, but there are differences between different groups. In *T. horrida* JY-521, *ThCYP14* has the highest number of introns, while *ThCYP20* only has one intron and the *ThCYP* genes may have experienced loss and increase of exons and introns during evolution. In this study, we predicted the subcellular localization of ThCYP proteins and found that they are located in different subcellular structures, including plasma membrane, cytoplasm, nucleus, mitochondria, and extracellular space. There are four ThCYP proteins in the plasma membrane, indicating that these proteins may be closely related to the membrane structure and intercellular interactions of cells. Four ThCYP proteins are located in the cytoplasm, indicating that these proteins may play a role in regulating cellular physiological functions and ensuring normal cell growth and metabolism. Three ThCYP proteins are located extracellular, indicating that these proteins may be secreted or transported to the outer side of the cell membrane. Eight ThCYP proteins are located in mitochondria, indicating that these proteins may be closely related to the structure and metabolic activity of mitochondria. ThCYP11 is located in the nucleus, indicating that it may be closely related to the structure of the nucleus and the processing of genetic information.

Cis-regulatory elements typically determine gene function by binding to specific transcription factors located upstream of genes. The CAREs in the promoter region significantly affect the precise function and regulation of genes [39]. Various CARE components related to developmental processes, defense, plant hormones, and stress responses were discovered in the promoter regions of *ThCYPs*. We detected multiple CAREs related to light responsiveness, such as G-box, Sp1, and GT1 motif, and identified growth-related CAREs, such as O2-site, RY-element, and CAT-box. In addition, we also identified plant hormones related to CAREs, such as MeJA response element (CGTCA motif), abscisic acid response element (ABRE), and salicylic acid response element (TCA element). MeJA and ABRE reaction elements are commonly present in the promoter regions of *ThCYPs*. In addition, we also detected CAREs (LTR, ARE, and GC motif) associated with multifactorial stress responses, which typically play a role in low-temperature responsiveness, body defense, and stress responsiveness. Some researchers have demonstrated that many cis-regulatory elements play crucial roles in multiple gene families [40,41]. In addition, it has been reported that the cis-regulatory elements of ABREs are associated with abiotic stress responses [42,43]. Currently, there are few cis-regulatory elements analyzed and reported in fungi. These explored and speculated fungal regulatory elements will help researchers accelerate the discovery of functional genes related to fungal development, fungal adaptability, and pathogenic processes.

We conducted PPI analysis on the ThCYPs to investigate their potential signaling pathways in RKS infection and expansion. We speculate that these proteins may play an important role in the infection timing of RKS infection. The PPI network analysis shows that the core proteins of the interaction include ThCYP2, ThCYP20, C-24 sterol reductase, fungal lanosterol synthase, and squalene monooxygenase, which are closely related and influence each other, forming the core of the interaction network. C-24 sterol reductase is involved in the fungal sterol biosynthesis pathway and regulates the structure and function of fungal cell membranes [44,45]. It plays an important role in regulating the synthesis and metabolism of sterol components in fungal cell membranes and has a significant impact on the biological characteristics and pathogenicity of fungi. Fungal lanosterol synthase plays an important regulatory role in fungal metabolic pathways, participating in the synthesis of fungal secondary metabolites, regulating fungal infection of hosts, and affecting fungal growth and survival [46,47]. It has a significant impact on the biological characteristics and pathogenicity of fungi. In addition, fungal squalene monooxygenase participates in the synthesis of fungal secondary metabolites and may play an important role in fungal infection of hosts.

CYPs are widely distributed in the genomes of fungi, bacteria, insects, plants, and mammals [11,31]. Some researchers have used CYP enzyme inhibitors to indirectly study the role of CYP enzymes in the degradation of heterologous compounds [48,49]. Many inhibition experiments have confirmed that fungal CYPs are crucial for biodegradation and play a crucial role in fungal xenobiotic detoxification [34]. Fungal CYP proteins can mediate the catalytic degradation of various environmental organic pollutants, and their expression is often induced by these exogenous compounds. The CYPs of lignin-degrading fungi are involved in the biodegradation of various endocrine disruptors [50]. The molecular evolution of fungal CYPs is at least partially driven by survival strategies, with the aim of providing a superior metabolic system to degrade exogenous chemicals [34]. The GO analysis results of this study showed that the function of *ThCYP* genes is mainly related to the metabolic process, participating in the sterol metabolic process, steroid metabolic process, alcohol metabolic process, organic hydroxy compound metabolic process, and lipid metabolic process. These results are consistent with previous research findings and provide information for further elucidating the role of *ThCYP* genes in the environmental adaptability and survival ability of fungi.

In summary, these findings provide conditions for a deeper understanding of the potential roles of *ThCYP* genes. Whole genome analysis enables us to preliminarily characterize the function of *ThCYPs*. It should be pointed out that the results obtained in this study are mainly based on computer analysis, and the true role of ThCYPs in pathogenicity still needs more experiments to verify. The function and molecular mechanism of *ThCYP* genes also need further exploration. The subsequent response of *T. horrida* after expressing these genes is currently unclear. We need to further determine which trigger genes in the host interact with *ThCYPs*. Heterologous expression of recombinant proteins is a good method for validating fungal CYP function. Developing an efficient and stable CYP heterologous expression system will greatly enhance our understanding of fungal CYP catalytic mechanisms and may lay the foundation for the potential practical application of CYP in bioremediation.

## 4. Materials and Methods

### 4.1. Identification and Phylogenetic Analysis of ThCYP Family Genes

In order to identify the CYP family genes in *T. horrida* JY-521, genomic data and annotation files were obtained from public genome sequencing databases based on information from previously published papers [22]. The physicochemical properties of ThCYP proteins, such as molecular weight, isoelectric point and GRAVE, were predicted using online ExPASy website (https://web.expasy.org/protparam/ accessed on 23 September 2024) [51]. Tools on the PSORT website were used for subcellular localization of proteins (https://wolfpsort.hgc.jp/ accessed on 23 September 2024).

### 4.2. Multiple Sequence Alignment and Phylogenetic Analysis

CYP family protein sequences from *T. horrida* JY-521 were aligned using Clustal W and default parameters. Based on multiple sequence alignment, a phylogenetic tree was constructed using the maximum likelihood (ML) method in MEGA 5.0, with a bootstrap value set to 1000.

### 4.3. Gene Structure, Protein Motif, and 3D Structure

GSDS (https://gsds.gao-lab.org/index.php accessed on 23 September 2024) was used to predict the exon–intron of *ThCYP* genes [52]. The MEME website (http://meme-suite.org accessed on 23 September 2024) was used to perform conservative motif prediction of *ThCYP* sequences, with a pattern count of 10 and other parameters set to default values [53]. The 3D structures of ThCYPs were created using the Phyre2 web server (http://www.sbg.bio.ic.ac.uk/phyre2/html/page.cgi?id=index accessed on 23 September 2024).

### 4.4. Promoter Cis-Acting Regulatory Elements (CARE) and Gene Ontology (GO) Analysis

The promoter sequences of 2000 bp upstream of the transcription start site of *ThCYP* genes were obtained from the *T. Horrida* JY-521 genome [22]. CAREs were identified using the PlantCARE online network server (http://bioinformatics.psb.ugent.be/webtools/plantcare/html/ accessed on 23 September 2024). The website (https://www.omicshare.com/tools accessed on 23 September 2024) was used for GO function enrichment analysis.

### 4.5. Protein–Protein Interactions

Online tools (https://cn.string-db.org/ accessed on 23 September 2024) were used to determine the protein–protein interactions among CYP family proteins and other partner proteins. The option of organisms on this website was set to *Ustilago maydis*. The website (https://www.omicshare.com/tools accessed on 23 September 2024) was used for functional annotation of CYP family proteins.

### 4.6. Analysis of Expression Patterns of ThCYP Family Genes

To investigate the expression of *ThCYP* genes at different infection time points, the RNA-seq data of ThCYP genes was collected from previous reports [18,21]. The website (https://www.omicshare.com/tools accessed on 23 September 2024) was used for drawing the heatmap based on the FPKM values.

### 4.7. RT-qPCR Analysis

According to the manufacturer’s instructions for the TIANGEN DP441 Reagent Kit (Tiangen, Beijing, China), we extracted total RNA from the fresh samples. The HiScript III 1st Strand cDNA Synthesis Kit (Vazyme, Nanjing, China) was used for the synthesis of first-strand complementary DNA (cDNA). Specific primers for each *CYP* gene were designed using Primer Premier 5.0 software. We performed qPCR using the LightCycler 96 PCR detection system (Roche, Basel, Switzerland). The *UBQ* gene was used as a reference gene to standardize the data, and the 2^−∆∆Ct^ method was used to calculate the relative expression level (Table S4).

## 5. Conclusions

In summary, this study provides a theoretical basis for exploring the gene functions and molecular mechanisms of *ThCYP* genes. ThCYPs are predicted to be functionally rich and may play an important role in the sterol metabolic process and the response to abiotic stress, deeply involved in pathogen–host interactions. Further research is also needed to determine whether the *ThCYP* genes have biological functions related to stress resistance. This study preliminarily analyzed the gene function of *ThCYP*s, laying the foundation for future functional research on other fungal *CYP* gene families.

## Figures and Tables

**Table 1:** Nomenclature and characteristics of the putative cytochrome P450 enzymes (CYPs) in *Tilletia horrida* that were detected.

Proposed Gene Name	Gene ID	Superfamily	Orientation	CDS Length (bp)	Protein Length (aa)	Mw (KDa)	PI	GRAVY	Predicted Subcellular Localization
*ThCYP1*	smut_0100	CYP51	Forward	1602	533	59.57	7.62	−0.264	mitochondrion
*ThCYP2*	smut_0267	CYP3	Reverse	1746	581	64.54	8.89	−0.142	plasma membrane
*ThCYP3*	smut_0564	CYP24	Reverse	1602	533	60.48	8.54	−0.375	cytosol
*ThCYP4*	smut_0646	CYP4	Forward	1701	566	63.23	8.45	0.031	mitochondrion
*ThCYP5*	smut_4482	CYP4	Forward	1872	623	68.57	6.64	−0.125	plasma membrane
*ThCYP6*	smut_1187	CYP3	Forward	1701	566	63.03	8.56	−0.033	mitochondrion
*ThCYP7*	smut_1646	CYP4	Reverse	1794	597	65.99	8.25	0.058	plasma membrane
*ThCYP8*	smut_2239	CYP4	Reverse	1707	568	63.15	9.14	−0.064	mitochondrion
*ThCYP9*	smut_2430	CYP46	Reverse	1821	606	67.60	9.44	−0.160	mitochondrion
*ThCYP10*	smut_2486	CYP4	Forward	1899	632	70.92	8.54	−0.101	cytosol
*ThCYP11*	smut_2490	CYP4	Reverse	1794	657	74.64	9.47	−0.227	nucleus
*ThCYP12*	smut_3024	CYP4	Forward	1914	637	70.38	8.66	−0.221	mitochondrion
*ThCYP13*	smut_3982	CYP3	Reverse	1797	598	67.29	6.78	−0.195	extracellular, including cell wall
*ThCYP14*	smut_4034	CYP4	Forward	1782	593	66.06	5.91	−0.094	plasma membrane
*ThCYP15*	smut_4035	CYP3	Forward	1716	571	64.19	7.25	−0.158	cytosol
*ThCYP16*	smut_4876	CYP4	Forward	1497	498	55.91	7.66	−0.262	mitochondrion
*ThCYP17*	smut_5517	CYP4	Reverse	1491	496	55.82	6.87	−0.178	extracellular, including cell wall
*ThCYP18*	smut_7145	CYP4	Forward	1731	576	64.56	9.08	−0.201	cytosol
*ThCYP19*	smut_6235	CYP2	Forward	3552	1183	130.99	6.11	−0.366	mitochondrion
*ThCYP20*	smut_6314	CYP5	Forward	1647	548	60.32	6.32	−0.082	extracellular, including cell wall

ID: identity; bp: base pair; aa: amino acids; PI: isoelectric point; Mw: molecular weight; GRAVY: grand average of hydropathicity; KDa: kilo dalton.

## Data Availability

The original contributions presented in the study are included in the article; further inquiries can be directed to the corresponding authors.

## Supplementary Materials

The following supporting information can be downloaded at: https://www.mdpi.com/article/10.3390/ijms251910478/s1.
