# Supplementary figures and images for: Whole Genome Identification and Biochemical Characteristics of the Tilletia horrida Cytochrome P450 Gene Family
Source: Int J Mol Sci. 2024 Sep 28;25(19):10478. doi: 10.3390/ijms251910478 (PMC11476942; doi:10.3390/ijms251910478)

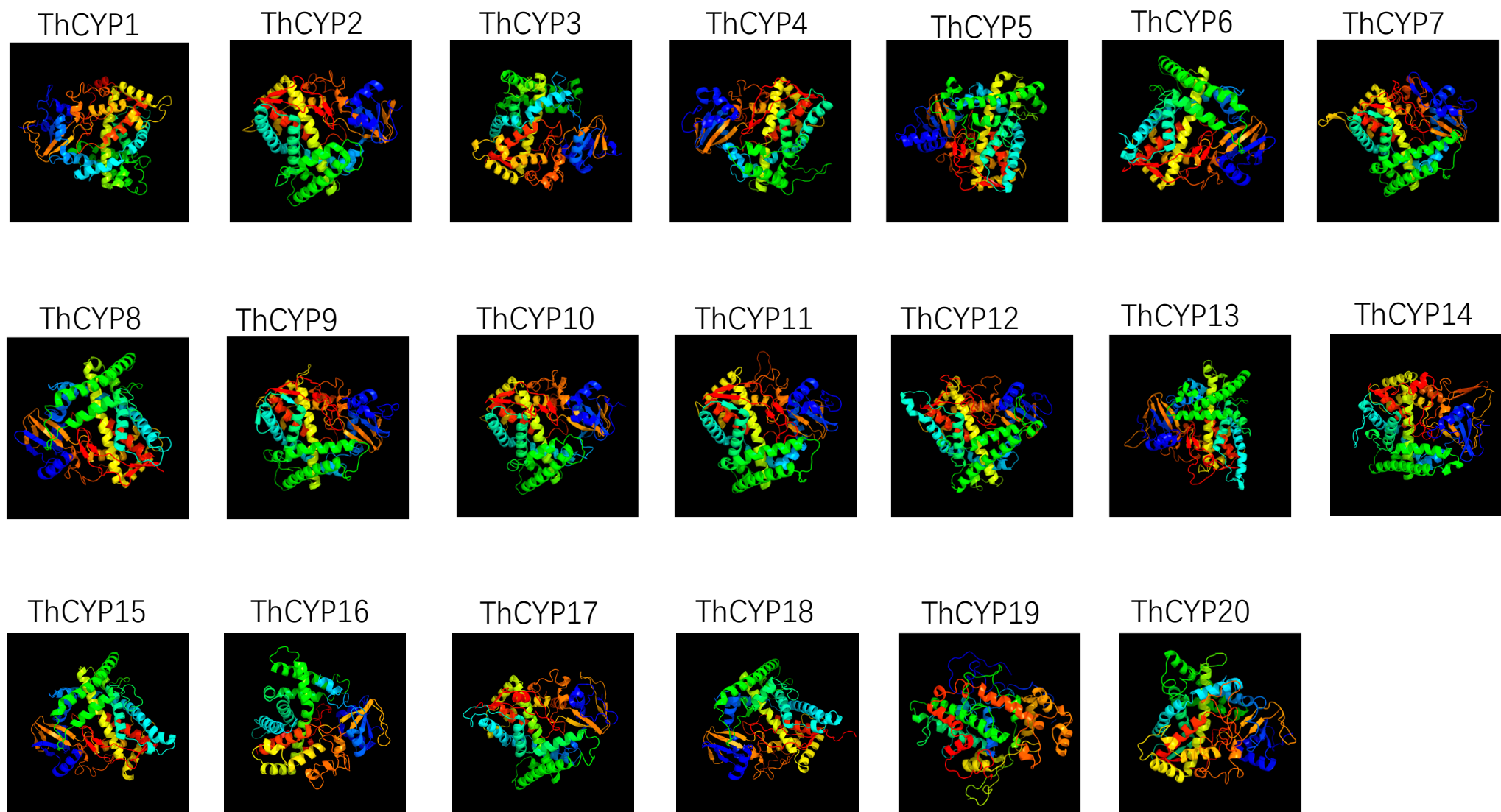

Figure S1. Predicted 3D structures of the ThCYP proteins.

Supplement: Supplementary file 1 [file ijms-25-10478-s001.zip › Figure.pdf]
